# Women view key sexual behaviours as the trigger for the onset and recurrence of bacterial vaginosis

**DOI:** 10.1371/journal.pone.0173637

**Published:** 2017-03-09

**Authors:** Jade E. Bilardi, Sandra M. Walker, Meredith J. Temple-Smith, Ruth P. McNair, Julie Mooney-Somers, Lenka A. Vodstrcil, Clare E. Bellhouse, Christopher K. Fairley, Catriona S. Bradshaw

**Affiliations:** 1 Central Clinical School, Monash University, Melbourne, Victoria, Australia; 2 Melbourne Sexual Health Centre, The Alfred Hospital, Melbourne, Victoria, Australia; 3 Department of General Practice, The University of Melbourne, Melbourne, Victoria, Australia; 4 Centre for Values, Ethics and the Law in Medicine, The University of Sydney, Sydney, New South Wales, Australia; 5 Department of Epidemiology and Preventative Medicine, Monash University, Melbourne, Victoria, Australia; 6 Melbourne School of Population and Global Health, The University of Melbourne, Melbourne, Victoria, Australia; Massachusetts General Hospital, UNITED STATES

## Abstract

**Background:**

Bacterial vaginosis (BV) affects a third of women of reproductive age in the US and there is increasing evidence to suggest it may be sexually transmitted. This study aimed to extend and validate the findings of our earlier smaller qualitative study by exploring in detail women’s views and experiences of the triggering factors associated with BV onset and recurrence.

**Methods:**

Women aged 20–49, who had experienced one or more symptomatic episode of BV within 6 months, were opportunistically recruited to complete a 38-item questionnaire on their experience of BV.

**Results:**

103 women completed the questionnaire. Women were significantly more likely to report sexual than lifestyle factors triggered BV onset and recurrence (p<0.001). The top 3 factors women attributed to both BV onset and recurrence were identical–and all sexual. They included, in order: 1) unprotected sex; 2) sex with a new male partner; and 3) sex in general. The main lifestyle factors nominated included stress, diet, menstruation and the use of feminine hygiene products. While many women felt *their BV* had been transmitted through sexual contact (54%) and developed as a result of sexual activity (59%), few considered BV a sexually transmitted infection (STI) (10%). Despite this 57% felt partners should also be treated for BV.

**Conclusion:**

These data concur with our earlier qualitative findings that women believe BV is triggered by sexual activity. While many women felt BV was sexually transmitted and supported partner treatment, they did not consider BV an STI. This contradiction is likely due to information conveyed to women based on current guidelines. In the absence of highly effective BV treatments, this study highlights the need for guidelines to indicate there is scientific uncertainty around the pathogenesis of BV and to contain clear health messages regarding the evidence for practices shown to be associated with a reduced risk of BV (i.e. consistent condom use.

## Introduction

Bacterial vaginosis (BV) is the most common vaginal condition affecting women of childbearing age, with between 10–30% of women in developed nations experiencing BV [1, 2]. Cross sectional studies also report a high prevalence of BV (20%-50%) among women who have sex with women (WSW) [3, 4]. Characterised by high loads of anaerobic bacteria including *Gardnerella vaginalis*, *Atopobium vaginae*, and non-cultivatable BV-associated bacteria (BVAB), and depletion of key *Lactobacillus* spp.[5], BV has been associated with serious sequelae including miscarriage, preterm delivery and increased risk of sexually transmitted infections (STI) and HIV [6–8]. Symptoms of BV commonly include a ‘fishy’ vaginal odour and an increased discharge [9, 10] which have been shown to significantly impact on women’s quality of life, in particular their self-esteem and sex lives [11].

While BV is highly prevalent among women, the aetiology and pathogenesis of this poly-microbial infection is not well understood and consequently it has been difficult to develop an effective treatment approach. Studies show up to 50% of women receiving recommended first line treatment for BV experience recurrence within 6–12 months [12, 13], however it is unclear whether high rates of recurrence are a consequence of treatment failure or reinfection from sexual partners [12, 14]. The role of sexual activity in the development of BV has been questioned for some time with increasing epidemiological and microbiological data suggesting BV may be sexually transmitted. Numerous studies have shown BV to be associated with sexual risk behaviours that typify STIs including: inconsistent or lack of condom use, new or multiple sexual partners, symptomatic female sexual partners, high frequency of sexual intercourse, young age at first sex and penile-vaginal sex [1, 2, 15–19]. A recent qualitative study undertaken with 35 Australian women with recurrent BV supported these findings, with the majority reporting BV onset had been triggered by sexual contact [20]. Microbiological data has also shown evidence of male-carriage of BV associated bacteria (BVAB), with uncircumcised male partners of females with BV showing a higher prevalence of BVAB than male partners of women without BV [21]. Concordant with this is evidence to show male circumcision is associated with a significant reduction of BVAB in men [22, 23] and a reduced risk of BV in women [24].

Women’s frustrations and embarrassment with the symptoms of BV often lead them to try a range of self-help remedies including douching, taking probiotics or vitamin supplements, using homemade suppositories or bath solutions, using over the counter vaginal treatments, avoiding tight clothing and perfumed soaps and modifying sexual and non-sexual lifestyle practices in an attempt to treat or prevent symptoms [25–31]. While some women report these remedies can be useful, most report little effect [31], with past research showing douching may be associated with a higher incidence of BV [1, 32–35] along with other ethnic and lifestyle factors including stress, the use of an intrauterine device, smoking, and non-white ethnicity [15, 36–39]. Currently, there is limited evidence to suggest probiotics, lactic acid or antiseptic and disinfectant treatments (e.g. hydrogen peroxide, chlorhexidine, povidone iodine) may be useful in the treatment of BV [40–42] and in a recent meta-analysis of observational data, hormonal contraception use was found to reduce the risk of BV but this remains to be evaluated by randomised controlled trial [43].

While published data indicates sexual activity plays a significant role in development of incident and recurrent BV, there is debate as to whether the aetiologic agent(s) of BV is transmitted through sexual activity or whether sexual activity simply disturbs the normal vaginal environment resulting in this vaginal dysbiotic state [44]. Current international guidelines [45] do not recommend partner treatment and women are frequently informed by their clinicians BV is not an STI [20]. Given the discordance between the epidemiological data, guidelines and clinical practice we sought to evaluate women’s views and experiences of the triggers associated with initial and recurrent episodes of BV. To our knowledge, this is the first survey based study to explore women’s experiences of the triggers for BV onset and recurrence. This study aimed to extend and validate the findings of our earlier smaller qualitative study [20] by exploring in detail women’s views and experiences of the triggering factors associated with BV onset and recurrence.

## Methods

### Ethics statement

Ethical approval for this study was granted by the Alfred Hospital Ethics Committee, Victoria, Australia; Application Number 488–14 on the 18^th^ December 2014.

### Study design

A self-administered 38-item BV questionnaire was completed by women over a one year period from February 2015 to January 2016 (S1 Appendix BV Questionnaire). Women had the choice of completing the questionnaire either online or in hard-copy. The questionnaire was informed by the findings of our previous qualitative study, current literature and the clinical expertise of the research group and included questions on: demographics, symptoms and diagnosis of BV, women’s views and knowledge of BV, the causes and triggers of first and recurrent episodes of BV, their use of self-help remedies, their opinions on the transmission of BV and the acceptability of possible preventative and treatment options for recurrent BV. To be eligible for the study women had to be aged 18–52 years, have experienced one or more diagnosed episode of BV in the past 6 months, be pre-menopausal and live in Victoria, Australia. Women under 18 years of age or over 52 years of age, who were menopausal, pregnant or living outside of Australia were excluded from the study.

### Cognitive interviewing to pre-test questionnaire

Prior to commencing the study, the questionnaire was pre-tested using cognitive interviewing (CI) methods. Traditional pretesting methods have assumed that if questionnaires are well designed, worded and pretested on a small number of people then flaws in question design can be identified and rectified ensuring results will be reliable and valid [46]. Traditional pretesting methods, however, do not take into account the ways in which different respondents understand and interpret the questions, whether all respondents interpret the questions in the same way and whether they are able to actually answer the questions [46]. Respondents will often answer survey questions even if they have not necessarily understood the question or are unsure of the meanings of words or ideas [46]. CI allows the researcher to address potential navigational issues and question-response problems so that respondents in the main study understand the question in the way they were intended and are able to answer the questions easily and with improved accuracy [47, 48].

A popular cognitive interviewing technique is verbal probing, which we conducted as part of the cognitive interviews. Verbal probing can either be used during the interview as the participant completes the questionnaire (concurrent probing) or after the participant has completed the questionnaire (retrospective probing) [49]. Probes may be *anticipated* i.e. written prior to the interview and asked of all participants if there are potential issues identified with questions or terms or *reactive*, allowing for flexibility as they are asked when unexpected difficulties in understanding arise during the interview [48]. One of the main strengths of CI’s is that they allow the interviewer to discuss issues of interpretation with participants, and change and test revised questions in further rounds of CI [48].

#### Cognitive interview iterations

For the purposes of this study, and with practical time limitations to consider, we conducted six CIs, with four questionnaire iterations in total. The first iteration included two concurrent interviews testing the hard copy questionnaire; the second and third iterations included one retrospective interview, per iteration, testing revised versions of the hard copy questionnaire. The third and final iteration included two retrospective interviews testing the ease of use and navigation of the online questionnaire. Of the six interviews, two were conducted via Skype, one was conducted by telephone and three were conducted face to face at MSHC. The pre-testing results and changes made to the questionnaire as a result are outlined in S2 Appendix Cognitive Interviewing.

### Recruitment

The main site for recruitment of women was the Melbourne Sexual Health Centre (MSHC), the largest sexual health clinic in Victoria, Australia. A small number of general practices and a family planning practice in metropolitan and regional Victoria, Australia, were also asked to refer eligible women. At all recruitment sites women were opportunistically invited to take part in the study by a clinician or nurse when they presented for a consult. Posters and leaflets were also placed in clinic waiting rooms with a study free- call phone number and website address to allow interested women to contact investigators directly or register their interest via the website. The BV Quest website (www.mshc.org.au/bvquest) provided information about BV in general, as well as information about the study and participant involvement. The study was explained in detail to women who contacted or were referred to the study and their eligibility and preference for questionnaire format confirmed. Depending on women’s preference of format, they were either posted a study pack including a plain language statement (PLS), consent form, hard copy of the questionnaire and a reply paid envelope or emailed a secure link to the online questionnaire which prevented unsolicited user access. The online questionnaire was hosted by REDCap, a password protected secure web based application to support data capture for research studies, which is secured and maintained by The Alfred Hospital and only accessible to study researchers [50]. Women using the online version were required to read the PLS prior to completing the questionnanire and indicate via a tick box their consent to participate. A total of three attempts were made to contact women interested in the study before they were deemed lost to follow up.

### Data analysis

Questionnaire data were extracted from REDCap and analysed in SPSS 23.0 using descriptive and frequency analysis. The chi square test was used to analyse categorical data including differences in the characteristics of participants who completed the questionnaire versus participants who did not complete the questionnaire. Questionnaire data was considered incomplete and excluded from the analysis if women had completed < 75%, including questions relating to the triggers for BV onset or recurrence. Questionnaires were considered complete and included in the analysis if ≥75% of questions were complete, including questions relating to the triggers for BV onset or recurrence. The Wilcoxin signed-rank test was used to test for equality between number of lifestyle factors and sexual risk factor responses for first and recurrent episodes of BV.

## Results

Of the 173 eligible women interested in the study, a total of 49 did not complete the questionnaire after consenting to participate in the study. Of the remaining 123 women, 20 did not meet the ≥75% completion requirements. Questionnaire data from 103 women were included in the study. 10 questionnaires were completed in hard copy format and 93 online. In comparing the demographic characteristics of the 20 women who did not complete the questionnaire to the 103 women who did, we found no statistically significant difference by age (≤25years vs >25 years) education level (university educated vs non university educated), place of birth (born in Australia vs born overseas), sexual identity (heterosexual vs non-heterosexual) or relationship status (in regular relationship vs not in regular relationship) [p>0.14].

### Participant characteristics

Table 1 outlines the demographic characteristics of participants. Nearly all participants were referred from MSHC and nearly two-thirds were born overseas and university educated. Most women (79%) identified as heterosexual and just over half were in a regular relationship, predominantly with a male partner. Of the participants, just over one-tenth had ever been paid for sex.

**Table 1:** Recruitment site and participant characteristics (n = 103).

	N (%*)	(95% CI)
**Recruitment site**		
MSHC	100 (97)	(92,99)
GP clinics/Family Planning Victoria	3 (3)	(0.6,8)
**Median age [range]**	25 [20–49]	
**Born in Australia**	37 (36)	(27,46)
**Education level**		
Up to Year 12	11 (11)	(5,18)
TAFE diploma or trade certificate	24 (23)	(16,33)
Undergraduate degree	47 (46)	(36,56)
Post graduate certificate or degree	19 (18)	(11,27)
Other	2 (2)	(0.2,7)
**Sexual identity**		
Heterosexual	81 (79)	(69,86)
Lesbian (queer)	9 (9)	(4,16)
Bisexual	10 (10)	(5,17)
Another identity (pansexual/demi-sexual/asexual)	3 (3)	(0.6,8)
**Gender identity**		
Female	101 (98)	(93,100)
Another identity (^^^gender fluid, trans non-binary)	2 (2)	(0.2,7)
**Ever been paid for sex**		
No	91 (88)	(81,94)
Yes	12 (12)	(6,19)
**Regular relationship**		
No	50 (49)	(39,59)
Yes	53 (52)	(41,61)
**Sex of partner**		
Male	48 (91)	(37,57)
Female	4 (8)	(1,10)
Transgender (female born)	1 (2)	(0.0,5)

*May not total 100% due to rounding up.

^ Gender fluid, trans non-binary: do not identify as either binary gender of male/female.

### Symptoms & diagnosis of BV

Table 2 outlines participants’ episodes, diagnoses and symptoms of BV. Over half of women had experienced more than one episode of BV (recurrence) and nearly all women experienced symptoms of vaginal odour and discharge when they had BV. The majority of women (86/103, 84%; 95% CI 75,90) had experienced their most recent episode of BV (first episode or recurrence) within the past month and nearly all women (n = 95, 92%; 95% CI:85,97) correctly related BV to a bacterial infection.

**Table 2:** Episodes, diagnoses and symptoms of BV (n = 103).

	N (%*)	(95% CI)
**Single episode BV**	42 (41)	(31,51)
**Recurrent episode/s BV**	61 (59)	(49,69)
**Median number [range] of separate**^^^ **episodes of BV**	2 [1–50]	
**Median number [range] of diagnosed**^**#**^ **episodes of BV**	1 [1–15]	
**Median age [range] of first diagnosis**	24 [13–49]	
**Symptoms usually experienced by women when they have BV:**		
Vaginal odour	93 (90)	(83,95)
Vaginal discharge	82 (80)	(71,87)
Other symptoms (itching, pain, burning)	14 (14)	(8,22)
No symptoms	2 (2)	(0.2,7)
**Most recent episode of BV**		
I have BV now	54 (52)	(42,62)
Within the past month	33 (32)	(23,42)
1–3 months ago	14 (14)	(8,22)
4–6 months ago	2 (2)	(0.2,7)

*May not total 100% due to rounding up.

^^^ Women were informed ‘separate’ episodes meant “*Symptoms have gone away following treatment or on their own and the next time you got BV you felt it was a new episode”*.

^**#**^ Women were asked “*How many times have you been diagnosed with BV by a doctor*?”.

### Triggers for first and recurrent episodes of BV

#### BV Onset

**Fig 1:**
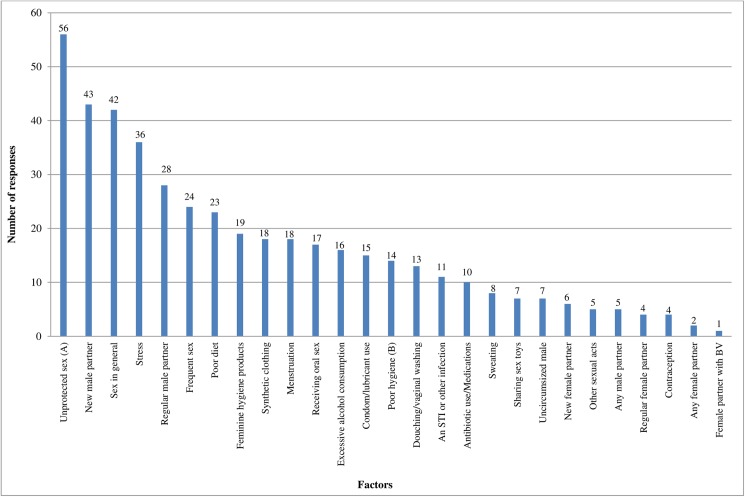
Factors women think caused or triggered BV onset (n = 452*). *n = 452—total number of responses 103 women who had experienced BV gave. Participants could choose multiple options. ‘*I do not know*’ responses (n = 4) were not included. (A)Unprotected sex with or without ejaculation. (B)Poor genital hygiene/unclean hands.

As part of the questionnaire, women were asked what they thought may have triggered or caused their first episode of BV (BV onset). Women were presented with a list of possible lifestyle and sexual factors and able to nominate in free text any other factors that were not listed. The top 3 factors women attributed to both BV onset and recurrence were identical–and all sexually related. In order, they included: 1) unprotected sex 2) sex with a new male partner 3) sex in general. The top 3 lifestyle factors women reported were: 1) stress 2) poor diet 3) feminine hygiene products. Fig 1 outlines, in descending order, the sexual and lifestyle factors women think triggered or caused BV onset.

Overall 81% (83/103; 95% CI: 72,88) of women nominated at least one lifestyle factor and 86% (89/103; 95% CI: 78,92) of women nominated at least one sexual factor triggered BV onset (women could choose multiple responses). We performed a Wilcoxon signed-rank test to test for equality between number of lifestyle and sexual factors for BV onset and found that women were significantly more likely to choose sexual versus lifestyle factor responses (p<0.001). Table 3 provides a summary of the parameter data for sexual versus lifestyle factor responses of women for both BV onset and most recent recurrence of BV.

**Table 3:** Summary of the parameters for the number of sexual and lifestyle factor responses nominated by women for BV onset and most recent recurrence.

BV onset			Most recent recurrence of BV		
	Lifestyle	Sexual		Lifestyle	Sexual
**Mean**	1.83	2.54		1.0	1.43
**Median**	2	2		0	0
**Range**	0–6	0–9		0–6	0–9
**IQR**	1–3	2–3		0–1	0–3

To determine if there were any demographic differences among women in terms of their sexual versus lifestyle factor responses, we compared the number of women who nominated *more* sexual factors (n = 60) with the number of women who nominated *more* lifestyle factors (n = 28) by demographic characteristics (88/103, 85%; 95% CI:77,92) the remaining women nominated an equal number of sexual and lifestyle factors and were excluded from the analysis). There was no significant difference by age, place of birth, education level, sexual identity, regular relationship or single versus recurrent BV (p>0.10), between women who chose more sexual risk factors versus women who chose more lifestyle factors for BV onset.

Of the 103 women, 38 (37%; 95% CI: 28,47) women reported symptoms of BV onset started within 7 days of the nominated factors or behaviours, 8 (8%; 95% CI: 3,15) within 8–14 days, 11 (11%; 95% CI: 5,18) more than 14 days later and 46 (45%; 95% CI: 35,55) reported they ‘did not know’ when their symptoms started.

#### Recurrence

**Fig 2:**
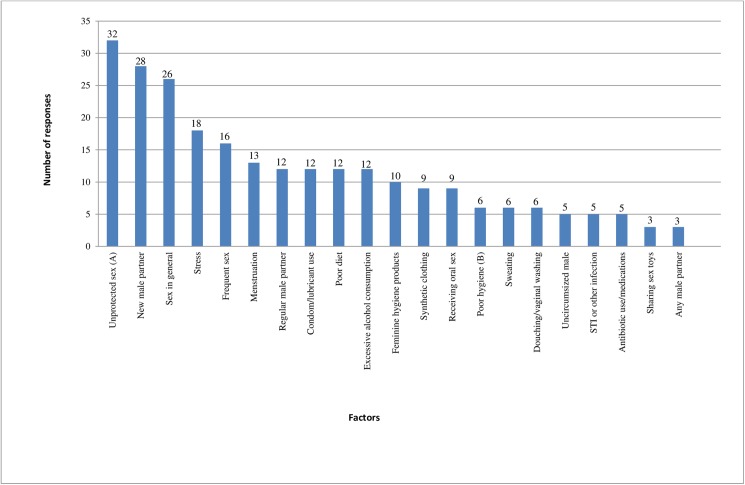
Factors women think triggered their most recurrent episode of BV (n = 248*). *n = 248—total number of responses 54 women who had experienced BV gave. Participants could choose multiple options. Did not include ‘I do not know’ responses n = 2. (A)Unprotected sex with or without ejaculation. (B)Poor genital hygiene/unclean hands.

Women who had experienced more than one episode of BV (n = 61) were also asked what they thought may have caused or triggered their most recent recurrence of BV. Once again, the top 3 factors were all sexual and the same factors as women nominated as triggering BV onset. The top 3 factors were: 1) unprotected sex 2) sex with a new male partner 3) sex in general. The top 3 lifestyle factors women reported for most recent recurrence were: 1) stress 2) menstruation 3) poor diet and excessive alcohol consumption. Fig 2 outlines in descending order the factors women think triggered or caused their most recent recurrence of BV (n = 54/61; *7 missing values).

Overall 43/54 (80%; 95% CI: 66,89) women nominated at least one lifestyle factors and 46/54 (85%; 95% CI 73,93) women nominated at least one sexual factors triggered their most recent recurrence of BV (women could choose multiple options of either). We performed a Wilcoxon signed-rank test to test for equality between number of lifestyle and sexual risk responses for most recent recurrence of BV and found that women were significantly more likely to choose sexual versus lifestyle risk responses (p = 0.02) to be associated with the onset of their most recent recurrence of BV (see Table 3 for a summary of the parameter data).

Again, to determine if there were any demographic differences among women in terms of their sexual versus lifestyle factor responses, we compared the number of women who nominated *more* sexual factors (n = 31) with the number of women who nominated *more* lifestyle factors (n = 15) (46/54; 85%; 95% CI: 73,93]; the remaining women nominated an equal number of sexual and lifestyle factors and were excluded from the analysis). As before, there was no significant difference by age, place of birth, education level, sexual identity, regular relationship or single episode versus recurrent BV (p>0.10), between women who chose more sexual risk factors versus women who chose more lifestyle for their most recent recurrence of BV.

Of the 54 women, 31 (57%; 95% CI: 43,71) of women reported the symptoms of their most recent recurrence started within 7 days of the nominated factors or behaviours, 7 (13%: 95% CI:5,25) within 8–14 days, 2 (4%: 95% CI: 0.5,13.0) more than 14 days later and14 (27%: 95% CI: 15,40) reported that they ‘did not know’ when their symptoms started.

### Transmission of BV

Interestingly, when asked “*Do you think your BV is transmitted through sexual contact*?” over half of women responded ‘*Yes*’ (55/102, 54%; 95% CI:43,63), while one third reported ‘*I do not know’* (n = 31, 30%; 95% CI: 21,40), and 16% (n = 16; 95% CI 9,24) responded ‘*No*’ (n = 102/103; *1 missing value). However, in the following question, only 10% (n = 10; 95% CI:5,17) reported they though BV was “*a sexually transmitted infection*”, with most responding ‘*BV develops because of sexual activity but is not an STI*’ (n = 60, 59%; 95% CI:48,68) and the remaining third of women responding either “*I do not understand why BV develops*” (n = 22, 22%; 95% CI:14,30) or offering alternative reasons via free text (n = 10, 10%; 95% CI:5,17) including: poor diet or immune system, excessive alcohol intake, poor gut health or the use or soap products. When asked about recurrences, most women (33/54, 61%; 95% CI: 47–74) felt they were “*A’flare up’* (*BV is still in your system and something triggers it again*)”, while 7 women (13%; 95% CI:5,25) reported they were due to a new partner (*“Due to a new partner giving you a new BV infection”)*, 1 (2%; 95% CI:0.05,10) attributed it to their regular partner (*“Due to your regular partner giving it back to you”)*, 8 did not know (“*I do not know*”) (15%; 95% CI:7,27) and 5 offered alternative reasons via free text including: partners poor sexual hygiene, poor diet or ‘complicated’ (9%; 95%CI:3–20) (n = 54/61; *7 missing values).

### Self-help remedies & behaviour modification

Table 4 outlines the self-help remedies women reported using when they had an episode of BV to try to treat or manage their symptoms.

**Table 4:** Self-help remedies women use to treat or manage their symptoms of BV (n = 103).

	N (%)	(95% CI)
**Shower more frequently**	52 (51)	(40,60)
**Use an over the counter vaginal treatment**	20 (19)	(12,28)
**Use homeopathic remedies or take specific** vitamins (including acidophilus/ lactobacillus/probiotics, folic acid, vitamins and cranberry tablets)	17 (17)	(10,25)
**Douche** (including douching with water, soap and hydrogen peroxide)	12 (12)	(6,19)
**Insert homemade suppositories** (including the insertion of acidophilus capsules, hydrogen peroxide, yogurt, vitamin C tablets, garlic cloves, tampons soaked in tea tree oil, coconut oil, apple cider vinegar)	11 (11)	(5,18)
**Use feminine hygiene products** (including deodorant sprays, soaps etc.)	11 (11)	(5,18)
**Treatment bath** (including salt water, apple cider vinegar, coconut oil and tea tree oil)	8 (8)	(3,15)
**Other** (including wearing cotton underwear, washing outside vagina after toileting, use/change panty liners regularly, sleep without underwear)	6 (6)	(2,12)

Table 5 outlines the sexual and lifestyle behavior’s women have stopped since first having BV, to try to prevent further recurrences.

**Table 5:** Sexual and lifestyle changes to prevent further recurrences of BV (n = 103).

*I no longer*:	N (%)	(95% CI)
**Have unprotected sex~**	28 (27)	(19,37)
**Use feminine hygiene products**	26 (25)	(17,35)
**Have sex with multiple partners**	17 (17)	(10,25)
**Receive oral sex**	16 (16)	(10,24)
**Wear tight or synthetic clothing**	16 (16)	(10,24)
**Have sex with casual partners**	13 (13)	(7,21)
**Douche**	13 (13)	(7,21)
**Have digital sex**	12 (12)	(6,20)
**Share sex toys with my partner**	7 (7)	(3,13)
**Other action or behaviour** (including no longer wearing daily panty-liners, no anal sex, no longer has sex with partner she believes triggered BV, makes partner wash hands/penis if dirty).	4 (4)	(1,10)

~Unprotected sex with or without ejaculationSome women also indicated they had also implemented other long-term lifestyle changes to try to prevent recurrences, including “*I have improved my diet”* (n = 30, 29%; 95% CI: 21,39), *“I now exercise/I exercise more regularly now”* (n = 20, 19%; 95% CI: 12,28), *“I have reduced my alcohol consumption”* or stopped smoking (n = 20, 19%; 95% CI: 12,28), *“I now take homeopathic remedies*” or *“I now take specific vitamins”* (n = 13, 13%; 95% CI: 7,21). Seven women also indicated via free text they had made other changes (7%; 95% CI: 3,14) including using pH balanced vaginal washes, improving genital hygiene, using certain brands of condoms only or using condoms/ using condoms more consistently.

### Partner treatment

When asked their views on partner treatment for BV, over half of women felt sexual partners should be treated for BV at the same time as them (n = 58/102, 57%; 95% CI:46,66) (includes ‘*yes*, *but only if they are male*’ [n = 1], *‘yes*, *but only if they are female’* [n = 15] and *‘yes*, *whether they are male or female* [n = 42]) (n = 102; *1 missing value). Of the women with a current regular partner, over half (27/51, 53%; 95% CI: 38,67) felt their partners would agree to being treated, 15 (29%; 95% CI: 17,44) did not know and 9 (18%; 95% CI: 8,31) did not think their partner would agree to being treated (n = 51; *2 missing values).

## Discussion

The findings from this study suggest women view BV onset and recurrence to be associated with sexual activity. Women were significantly more likely to report sexual rather than lifestyle factors triggered BV onset and recurrence. The top 3 factors women attributed to both BV onset and recurrence were identical–*and all sexual*. In order, they included: 1) unprotected sex 2) sex with a new male partner and 3) sex in general. These findings concur with our earlier qualitative study [20], which also found the majority of women felt sexual activity triggered BV onset, the main triggers including unprotected sex and sex with a new male partner. Epidemiological data strongly supports women’s experiences of the sexual triggers for BV, with studies commonly finding an association between an increased risk of BV and inconsistent or lack of condom use or a new sexual partner [2, 17, 51–55]. To a lesser extent, lifestyle triggering factors nominated by women, such as psychosocial stress, have also been associated with an increased risk of BV [39, 56].

Women’s beliefs around the triggers for BV were reflected in the sexual and lifestyle modifications they made to try to prevent further recurrences, with nearly a third of women reporting they no longer had unprotected sex with partners and others reporting the cessation of a variety of other sexual practices. The lifestyle triggers and modifications women reported, including long term dietary and exercise changes, are analogous with our previous findings [31], and also reflect generic advice given to women (to avoid douching, soap or feminine hygiene products) and women’s desire to improve their general health to reduce their susceptibility to BV. Women’s use of self-help remedies to treat symptoms of BV mirror those found in other studies examining women’s experiences of vaginal conditions or recurring STIs [25–31, 40, 57], however it is encouraging to see in this study only a minority of women reported douching, given its association with an increased risk of BV [1, 32–35].

Interestingly, while women commonly felt BV was associated with sexual contact, few actually think BV is an STI. In this study, we directly asked women if they thought *their BV* was *transmitted* through sexual contact, to which over half of women said yes, and yet, when asked next whether they thought BV was a STI or develops because of sexual activity but is not an STI, the majority chose the latter despite just previously indicating they thought *their BV* had been *transmitted* through sexual contact. This biological contradiction reflects the confusion in the medical field regarding the role of sexual transmission in the pathogenesis of BV and the difficulties faced by clinicians who may err on the side of making definitive statements rather than conveying any scientific uncertainty to their patients. Most of the women in this study were also recruited from the MSHC which routinely provides a handout to women diagnosed with BV which states sexually active women are more likely to get BV however it is not known if BV is sexually transmitted [58]. This contradiction also likely reflects women’s reluctance to think of BV as an STI due to the stigma and shame commonly associated with an STI diagnosis [20, 59, 60].

Despite not believing BV is an STI, over half of women in this study thought their partner should be treated for BV at the same time as them. Given the impact of recurrent BV on women’s sex lives and self-esteem [11] is it not surprising they are likely to support any new treatment approaches that may reduce their risk of recurrences. These data highlight the conflict women experience in their inherent views about the factors associated with BV onset and recurrence and the medical information given to them by professionals and the information available online. Our earlier qualitative study [11] found women commonly experienced high levels of distress, embarrassment and confusion as a result of having BV, which is likely to be driven by not only the high rates of recurrence following recommended antimicrobial therapies [12], but also due to the inadequacy of available clinical information which conflicts with the epidemiological data and their own views and experience of the condition.

### Strengths & limitations

The major strength of this study is that it is the first survey based study we are aware of, to explore in detail women’s experiences of the triggers and transmission of BV. A further strength of this study is that most women were recruited within one month of having an episode of BV and therefore it is likely their recall bias was limited.

Clinicians and researchers do not have the tools to differentiate between incident and recurrent BV and therefore we are unable to know whether women reporting recurrence were experiencing symptoms from a latent persistent infection or had acquired a new infection, which has important implications as the underlying aetiology and pathogenesis for these may differ. We hypothesize that a BV episode that occurs shortly after the first episode of BV is likely to reflect recurrence associated with either reinfection from a partner or re-emergence of persistent infection, whereas a subsequent BV episode which occurs a couple of years after a first episode of BV is likely to reflect a completely new incident infection. In future, it would be worth looking more closely at when recurrences have occurred and the triggers women attribute to recurrences depending on the time period they occurred within i.e. weeks/months/years after new incident BV.

A further limitation of this study is that women were presented with numerous structured options for possible sexual and lifestyle triggers, which included a slight imbalance between the number of sexual (n = 15) and lifestyle factors (n = 12) with three more sexual factors listed compared to lifestyle factors, which may have influenced women’s responses. In hindsight it would have been useful to ask women to nominate the single greatest factor they thought triggered BV onset and most recent recurrence. In offering women the option of nominating multiple trigger factors we were unable to analyse the data by number of women and analysed according to number of nominated responses, however, in stating this, it was important to allow women to select multiple options and overall we did find women nominated statistically more sexual risk factors compared to lifestyle factors. It is also important to acknowledge that nearly two-thirds of our sample was university educated and almost all women were recruited from one metropolitan sexual health clinic and therefore our results may not be generalisable to the broader population of women in the community, including from different geographic locations. Lastly, while our sample included a large proportion of non-heterosexual women, only a small number had female partners so there was insufficient power to compare responses with heterosexual women.

### Future implications

It is clear from this study that women’s experiences of the triggers for BV concur with mounting epidemiological and microbiological data which show sexual contact is associated with BV, and support the notion sexual transmission is likely to be playing a significant role in the acquisition and recurrence of BV. While many women feel BV is sexually transmitted and partners should be treated, they do not, however, classify it as an STI. This inconsistency is likely due to international treatment guidelines which inform practitioners on an evidence base from past male partner treatment trials which did not show consistent benefit in reducing BV recurrence in women [61]. Importantly two recent systematic reviews of these trials found significant flaws in trial design and ranked the evidence of a low to very low quality in terms of the effectiveness of partner treatment in reducing BV recurrence [62, 63]. The conflict between these trial findings and mounting microbiological and epidemiological data highlights the need for new well powered partner treatment trials to provide an accurate evidence base for future guidelines [62]. Importantly our study shows most women believe their partners should be treated for BV at the same time as them which means they are likely to be receptive to engaging in partner treatment trials. One such male partner treatment trial is currently underway in North America (ClinicalTrials.gov Identifier: NCT02209519) and further trials examining the use of combination treatment therapies are planned—we eagerly await the outcomes of these studies.

These data highlight the need for guidelines to indicate there is scientific uncertainty around the pathogenesis of BV and to include some evidence for practices that have been shown to be detrimental or associated with a reduced risk of BV. Our research has shown that women are frustrated and disheartened by the poor efficacy of recommended antimicrobials and confused by the inconsistency of medical advice and this leads them to seek alternative non-evidence based and largely ineffective therapies [31]. By providing women with information at the time of treatment regarding practices that have been consistently associated with increased risk of BV i.e. inconsistent or lack of condom use, sex with a female with BV or multiple partnerships, and conveying the need for further trials to establish if BV is sexually transmitted, it clarifies the current status of medical evidence for women which is an acceptable approach across all disciplines of medicine when there are still significant knowledge gaps.

## Supporting information

S1 Appendix BV Questionnaire. (PDF)

S2 Appendix Cognitive Interviewing. (DOCX)
