# Supplementary material for: Women view key sexual behaviours as the trigger for the onset and recurrence of bacterial vaginosis
Source: PLoS One. 2017 Mar 9;12(3):e0173637. doi: 10.1371/journal.pone.0173637 (PMC5344463; doi:10.1371/journal.pone.0173637)
Supplement: S1 Appendix — (PDF) [file pone.0173637.s001.pdf]

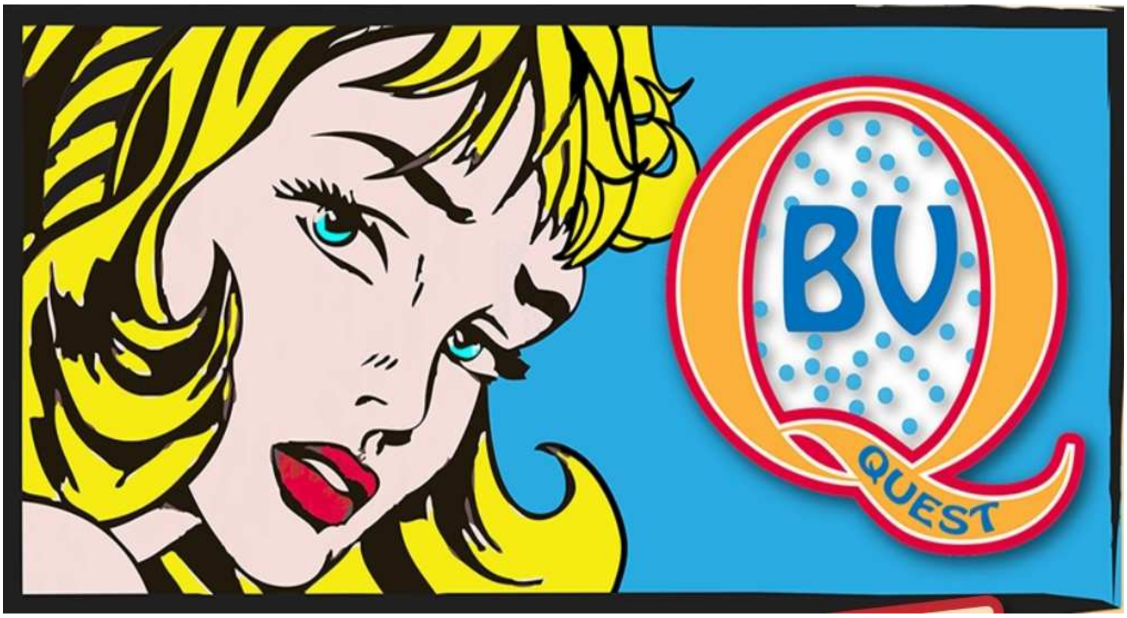

BV QUESTIONNAIRE

STUDY ID: ______________

Thank you for taking part in this study to tell us about your experience of bacterial vaginosis (BV). This questionnaire includes some sensitive questions. However, we do not ask you for any information that will identify who you are, and it is important to remember that any information you give us will remain confidential.

Please read the participant information statement for more details about this study. Please complete the following questionnaire, which will take approximately 25 minutes of your time. For some questions you will need to complete the text boxes or place a tick in the box of the answer that best fits your response. Unless directed otherwise (i.e. '*tick all that apply*') please tick one response box only.

Thank you once again for your participation. If you have any questions about the questionnaire or your participation in the study please contact our study investigators on free call 1800 234 285.

MONASH University | THE UNIVERSITY OF MELBOURNE | TheAlfred | Melbourne Sexual Health Centre — A service within The Alfred, a member of Bayside Health

DEMOGRAPHICS

1. Today's date ……/……/…… Age: …………… Postcode: ……………

2. Where were you born: ☐ Australia[1] ☐ Overseas[2]

3. What is your ethnic background? *(i.e. your parents birthplace/s)*……………………………………………………………

4. What is the highest level of formal education you have <u>completed</u>?

   ☐ Up to Year 10[1]
   ☐ Year 12/ VCE/HSC/QCE/SACE/WACE[2]
   ☐ Tertiary diploma or trade certificate/TAFE[3]
   ☐ Undergraduate university degree[4]
   ☐ Post graduate university degree[5]
   ☐ Other[6] – *Please specify* ……………………………………………

5. What is your current employment status? *(please tick all that apply)*

   ☐ Full time[1]
   ☐ Part-time or casual[2]
   ☐ Retired[3]
   ☐ Student[4]
   ☐ Unemployed[5]
   ☐ Other[6] – *Please specify* ………………………………………………………………………

6. Which of the following best describes your **<u>sexual identity</u>**? (*please do not feel limited to the suggestions below, we welcome your personal response to this item in the box 'Another identity'*)

   ☐ Heterosexual[1]
   ☐ Queer[2]
   ☐ Gay[3]
   ☐ Lesbian[4]
   ☐ Bisexual[5]
   ☐ Another identity[6] – *Please specify* ………………………………………………………

7. Which of the following best describes your **<u>gender identity</u>**? (*please do not feel limited to the suggestions below, we welcome your personal response to this item in the box 'Another identity'*)

   ☐ Female[1]
   ☐ Female to male transgender[2] - I have been through genital surgery ☐ No[0] ☐ Yes[1] ☐ Prefer not to say[2]
   ☐ Male to female transgender[3] - I have been through genital surgery ☐ No[0] ☐ Yes[1] ☐ Prefer not to say[2]
   ☐ Intersex[4]
   ☐ Another identity[5] - *Please specify* ……………………………………………………..

8. Have you ever been paid for sex?

   ☐ No[0]
   ☐ Yes[1] – When? ☐ I am currently paid for sex Months or Years worked ……………………….. *(i.e. May 2010-Now)*
   ☐ I am no longer paid for sex Months or Years worked ……………………….. *(i.e. June 2008-2010)*
   ☐ Other: please specify (*i.e. once off only*) ………………………………………………………………………

9. Where did you hear about our study?

   ☐ Melbourne Sexual Health Centre[1]
   ☐ Other sexual health clinic[2] – *Please specify* ………………………………………………………………
   ☐ GP clinic[3] – *Please specify* ……………………………………………………………………………………
   ☐ Family Planning Victoria[4]
   ☐ Other[5] – *Please specify* ………………………………………………………………………………………

10. Are you currently in a regular sexual relationship? (*with someone <u>you consider</u> to be a regular or ongoing sexual partner i.e. boyfriend/girlfriend/partner*)

    ☐ No[0] – go to Q12 ☐ Yes[1]

11. Is your regular sexual partner/s:

    ☐ Male[1]
    ☐ Female[2]
    ☐ Both - I have both a regular male and female partner[3]
    ☐ Other partner[4] – *Please specify*……………………………………………………………

SYMPTOMS & DIAGNOSIS OF BV

BV is often characterized by a strong, unpleasant, fishy smell and a thin, watery white/grey discharge.

12. What symptoms do you usually experience when you have BV? *(tick all that apply)*

- ☐ Unpleasant vaginal odour[1]
- ☐ Abnormal vaginal discharge[2]
- ☐ I do not experience any symptoms[3]
- ☐ Other symptoms[4] - *Please specify* ………………………………………………
…………………………………………………………………………………………

13. How many **separate*** episodes of BV *do you think* you have had? ……………

*(*Symptoms have gone away following treatment or on their own and the next time you got BV you felt it was a new episode).*

Please comment further if you would like ……………………………………………………………………………
……
……

14. How many times have you been *diagnosed* with BV by a doctor? ……………

Please comment further if you would like ……………………………………………………………………………
……
……

15. At what age do you think you first had BV? …………..

16. At what age were you *first diagnosed* with BV by a doctor? …………..

17. When do you think you last had BV?

☐ I have BV now OR ……….week(s) ago OR ……….month(s) ago OR ……….years (s) ago

VIEWS & KNOWLEDGE OF BV

We are now asking you about your knowledge of BV (please tick one option only).

18. I believe BV is…..

- ☐ A yeast/fungal infection[1]
- ☐ A bacterial infection[2]
- ☐ Not an infection[3]
- ☐ I do not know[98]

19. Which of the following *do you think* increases a womans risk of BV? (*tick all that apply*)

- ☐ Being overweight[1]
- ☐ Smoking[2]
- ☐ Pregnancy[3]
- ☐ Douching inside the vagina with water or other products[4]
- ☐ Using perfumed products *(i.e. soaps or feminine hygiene products)*[5]
- ☐ Poor diet[6]
- ☐ Sex with a new partner[7]
- ☐ Sex with a regular partner[8]
- ☐ Sex with a number of different sexual partners[9]
- ☐ Sex with a female partner with BV[10]
- ☐ Unprotected sex[11]
- ☐ Sex with an uncircumcised male[12]
- ☐ Other[13] – *Please specify* ……………………………………………………………………………
- ☐ None of the above[97]
- ☐ I do not know[98]

FIRST EPISODE OF BV

20. Can you tell me about your *__first episode__* of BV? *(Please answer the following dot points)*

- When did you first get BV?
- How long did your symptoms last?
- When did you seek medical assistance (*i.e. the next day, two weeks later etc.)*
- Did you go to a doctor for treatment and/or try to treat it yourself?

………
………
………
………
………
………
………
………
………
………
………

21. Thinking back to your *__FIRST episode of BV__* what do you think may have caused or triggered it? *(Please tick all that apply. Leave blank if none of the options apply or add your own under 'Other –Please specify')*

Lifestyle - My first episode of BV was caused or triggered by:

- ☐ Poor genital hygiene[1]
- ☐ Poor diet[2]
- ☐ Excessive alcohol consumption[3]
- ☐ Having an STI (such as chlamydia or herpes)[4]
- ☐ Perfumed products *(i.e. soaps or feminine hygiene products)*[5]
- ☐ Having my period (on or around that time)[6]
- ☐ Douching[7]
- ☐ Stress[8]
- ☐ Sweating[9]
- ☐ Antibiotic use[10]
- ☐ Tight or synthetic clothing[11]
- ☐ Medications[12]
- ☐ Other[13]– *Please specify*……………………………………………………………………………

Sexual partnerships- My first episode of BV was caused or triggered by:

- ☐ Sex with a new male partner[14]
- ☐ Sex with a new female partner[15]
- ☐ Sex with my regular male partner[16]
- ☐ Sex with my regular female partner[17]
- ☐ Sex with any male partner[18]
- ☐ Sex with any female partner[19]
- ☐ Sex with an uncircumcised male[20]
- ☐ Other[21]– *Please specify*…………………………………………………
  ……………………………………………………………………………

Sexual acts - My first episode of BV was caused or triggered by:

- ☐ Sex in general[22]
- ☐ Unprotected sex with ejaculation[23]
- ☐ Unprotected sex without ejaculation[24]
- ☐ Receiving oral sex[25]
- ☐ Sharing sex toys with my partner[26]
- ☐ Frequent sex[27]
- ☐ Using lubricant[28]
- ☐ Using condoms[29]
- ☐ Other[30]– *Please specify*…………………………………………………
  ……………………………………………………………………………

22. Approximately how long after these triggers or behaviours did your symptoms of BV start?

……..day(s) later OR ………..week(s) later OR ………..month(s) later ☐ I do not know how long

23. Please tell us more about why you think these factors may have triggered or caused your BV.

………
………
………
………
………

RECURRENT EPISODES OF BV

If you have experienced more than one episode of BV (recurrent BV) please complete...otherwise go to Q29

24. Can you tell me about your recurrent episodes of BV? *(Please answer the following dot points)*

- When does BV usually come back *(i.e. is there a pattern or a time when you are more likely to get it?)*
- Have the symptoms been similar or different to your first episode?
- How do you normally treat BV when it comes back?

....

....

....

....

....

....

....

....

....

....

....

25. Thinking about your most RECENT recurrence of BV what do you think may have caused or triggered it? *(Please tick all that apply. Leave blank if none apply or add your own under 'Other –Please specify')*

Lifestyle - My most recent episode of BV was triggered by:

- ☐ Poor genital hygiene[1]
- ☐ Poor diet[2]
- ☐ Excessive alcohol consumption[3]
- ☐ Having an STI (such as chlamydia or herpes)[4]
- ☐ Perfumed products *(i.e. soaps or feminine hygiene products)*[5]
- ☐ Having my period (on or around that time)[6]
- ☐ Douching[7]
- ☐ Stress[8]
- ☐ Sweating[9]
- ☐ Antibiotic use[10]
- ☐ Tight or synthetic clothing[11]
- ☐ Medications[12]
- ☐ Other[13]– *Please specify*..

Sexual partnerships- My most recent episode of BV was triggered by:

- ☐ Sex with a new male partner[14]
- ☐ Sex with a new female partner[15]
- ☐ Sex with my regular male partner[16]
- ☐ Sex with my regular female partner[17]
- ☐ Sex with any male partner[18]
- ☐ Sex with any female partner[19]
- ☐ Sex with an uncircumcised male[20]
- ☐ Other[21]– *Please specify*..
....

Sexual acts - My most recent episode of BV was triggered by:

- ☐ Sex in general[22]
- ☐ Unprotected sex with ejaculation[23]
- ☐ Unprotected sex without ejaculation[24]
- ☐ Receiving oral sex[25]
- ☐ Sharing sex toys with my partner[26]
- ☐ Frequent sex[27]
- ☐ Using lubricant[28]
- ☐ Using condoms[29]
- ☐ Other[30]– *Please specify*..
....

26. Approximately how long after these specific triggers or behaviours did your symptoms of BV start?

........day(s) later OR ....week(s) later OR ....month(s) later ☐ I do not know how long

27. Please tell us more about why you think these factors may have triggered your most recent recurrence.

....

....

....

....

....

28. Do you think that your recurrences of BV are: *(please tick one option only)*

- ☐ A 'flare up' *(i.e. BV is still in your system and something triggers it again)*[1]
- ☐ Due to your regular partner giving it back to you[2]
- ☐ Due to a new partner giving you a new BV infection[3]
- ☐ Because I don't always finish my course of antibiotics[4]
- ☐ Other[5] – *Please specify*...
- ☐ I do not know[98]

SECTION F: ALTERNATIVE TREATMENTS & PREVENTION

29. When you **HAVE BV** do you do any of the following to ***treat or manage your symptoms***? *(Please tick any that apply)*

	Please specify
☐ Shower more frequently[1]	
☐ Douche with water or soap inside your vagina[2]	....
☐ Use an over the counter treatment[3] *(i.e. Acid Gel Hydrosol, Vagil Clear)*	....
☐ Treatment bath[4] *(i.e. salt water, apple-cider vinegar bath etc.)*	....
☐ Insert homemade suppositories[5] *(i.e. yoghurt, vinegar soaked tampons)*	....
☐ Use homeopathic remedies[6]	....
☐ Take specific vitamins[7]	....
☐ Use feminine hygiene products[8] *(i.e. deodorant sprays, soaps etc.)*	....

☐ Other action or behaviour [9] - *Please explain* ....

30. Since getting BV, have you **STOPPED** doing any of the following to try and prevent recurrences? *(Please **do not tick** if you did not do these things before anyway)*

- ☐ I no longer receive oral sex[1]
- ☐ I no longer have unprotected sex[2]
- ☐ I no longer allow my sexual partners to ejaculate inside me[3]
- ☐ I no longer share sex toys with my partner[4]
- ☐ I no longer let my partners put their fingers or hands inside my vagina[5]
- ☐ I no longer have sex with a number of different sexual partners[6]
- ☐ I no longer have sex with casual partners[7]
- ☐ I no longer douche[8]
- ☐ I no longer use feminine hygiene products[9] *(i.e. deodorant sprays, soaps etc.)*
- ☐ I no longer wear tight or synthetic clothing [10]
- ☐ Other action or behaviour [11] - *Please explain* ....

**Please tick if you NOW do the following to prevent recurrences**	***Please specify***
☐ I now take homeopathic remedies[1]	....
☐ I now take specific vitamins[2]	....
☐ I have improved my diet[3]	....
☐ I now exercise / I exercise more regularly now[4]	
☐ I have reduced my alcohol consumption[5]	

☐ Other action or behaviour[6] - *Please explain*...

SEX & BV

Please answer Q31-33 <u>only if you have a current regular female sexual partner</u> otherwise go to Q34.

31. Have you had an episode of BV while you have been with your current female partner?

☐ No[0] ☐ Yes[1] ☐ I do not know[98]

32. Has your current female partner ever had BV around the same time as you (i.e. within 2 weeks)?

☐ No[0] ☐ Yes[1] ☐ I do not know[98]

33. Do you think your BV was transmitted between you and your partner through sexual contact?

☐ No[0] ☐ Yes[1] ☐ I do not know[98]

The cause of BV is still unknown. In your opinion:

34. Do you think your BV is transmitted through sexual contact? *(please tick one option only)*

☐ No[0] ☐ Yes[1] ☐ I do not know[98]

35. Do you think BV: *(please tick all that apply)*

☐ Is a sexually transmitted infection (STI)[1]
☐ Develops because of sexual activity but is not an STI[2]
☐ I do not understand why BV develops[3]
☐ Other[4]- *Please explain* ………………………………
……………………………………………………………

POSSIBLE PREVENTATIVE & TREATMENT OPTIONS

36. If you had a partner and you got BV, do you think they should be treated for BV at the same time as you? *(please tick one option only)*

☐ No – I do not think partners should be treated[0]
☐ Yes – but only if they are male[1]
☐ Yes – but only if they are female[2]
☐ Yes – whether they are male or female[3]
☐ Other[4] – Please specify……………………………………………………
☐ I do not know[98]

Please answer Q37 <u>only if you have a current regular male or female partner</u> otherwise go to Q38

There are a number of preventative and treatment options we would like to study to determine if they reduce the risk of BV coming back. These are based on recent research findings but have not been subject to a clinical trial. One possible treatment option is partner treatment which would involve both the patient and their partner being treated for BV to see if it reduces the risk of BV coming back. For female partners treatment would involve taking a 7-day course of oral antibiotics. For male partners treatment would involve taking a 7-day course of oral antibiotics and may also involve applying antibiotic cream on the penis daily.

37. Do you think your current regular partner would agree to be treated for BV at the same time as you?

☐ No[0] ☐ Yes[1] ☐ I do not know[98]

*If '**no**' or '**I do not know**', why not?*

………
………
………
………

*If '**yes**', do you think they would be more likely to take treatment if: (please tick all that apply)*

- ☐ They were required to see your doctor for treatment[1]
- ☐ They were required to see their doctor for treatment[2]
- ☐ You were able to take home treatment for them without them needing to see a doctor[3]
- ☐ They were required to have a phone consultation with your doctor who would send them treatment[4]
- ☐ You could give them a letter which they could take to any doctor with instructions on treatment action[5]
- ☐ Other[6] – Please specify………………………………………………………

38. Would you be willing to participate in a clinical trial to determine if the following reduces your risk of BV coming back:

a) Taking the hormonal contraceptive pill for 6 months (The Pill)

☐ No[0] ☐ Yes[1] ☐ Unsure[98]

If 'no' or 'unsure', why? ……………………………………………………………………………………………
………

b) Enrolling with **<u>your regular male partner</u>** in a partner treatment trial where you both take antibiotic treatment for BV

☐ No[0] ☐ Yes[1] ☐ Unsure[98] ☐ No male partner[97]

If 'no' or 'unsure', why? ……………………………………………………………………………………………
………

c) Enrolling with **<u>your regular female partner</u>** in a partner treatment trial where you both take antibiotic treatment for BV

☐ No[0] ☐ Yes[1] ☐ Unsure[98] ☐ No female partner[97]

If 'no' or 'unsure', why? ……………………………………………………………………………………………
………

<u>If you have a regular male partner</u> could you please ask them to complete the Male Partner Treatment Questionnaire either online (using the email links provided) or in your hard copy pack and return it with your questionnaire.

<u>If you have a regular female partner</u> could you please ask them to complete the Female Partner Treatment Questionnaire either online (using the email links provided) or in your hard copy pack and return it with your questionnaire.

Thank you for your time and input into this study. Your contributions are invaluable and very much appreciated. If you have any additional questions, Dr Jade Bilardi will be happy to answer them (Mob: 0402 724 228 or jbilardi@mshc.org.au). If you have any complaints about any aspect of the project, the way it is being conducted, or any questions about your rights as a research participant, you may contact Ms Rowan Frew, Manager, Office of Ethics and Research Governance, The Alfred, at R.Frew@alfred.org.au or by phone on (03) 9076 3848.
