# Supplementary material for: Women view key sexual behaviours as the trigger for the onset and recurrence of bacterial vaginosis
Source: PLoS One. 2017 Mar 9;12(3):e0173637. doi: 10.1371/journal.pone.0173637 (PMC5344463; doi:10.1371/journal.pone.0173637)
Supplement: S2 Appendix — (DOCX) [file pone.0173637.s002.docx]

**S2 Appendix: Cognitive interviewing pre-testing results and changes**

| **Ambiguity** | Using both anticipated and reactive probes we identified a number of terms including ‘*increased risk of BV’* and ‘*regular sexual partner’* that were not understood in the same way by all participants. The definitions were clarified in subsequent rounds and no further issues identified with these terms. The term ‘*ethnic background’* was also understood in different ways, with the first two participants questioning the appropriateness or meaning of the response categories provided. Further explanation of the term was provided in the question and close ended response categories replaced with open ended text response. |
| --- | --- |
| **Concepts & Comprehension** | One question on women’s use of self-help remedies proved particularly difficult for women to interpret accurately due to question wording and response options provided - ‘*When you do not have BV, do you do any of the following to specifically try and prevent further recurrences?’* The question and response options were redrafted and retested in questionnaire iterations two and three. The final question version was reworded to ask women - ‘*Since getting BV, have you STOPPED doing any of the following to try and prevent recurrences*?’ Women were asked not to tick the boxes of any actions or behaviours they had not done previously done. A further question that proved difficult to answer for many women was the number of times they had had BV in the past. Women were unsure how to define an individual ‘episode’ of BV as they commonly experienced episodes where symptoms subsided but never fully went away and then ‘flared up’ again at a later date. The question was reworded to ‘*separate*’ episodes of BV and a definition of ‘*separate episodes’* provided to assist women in defining single episodes. A free text space was also provided to allow for further explanation if necessary. Further free text was also inserted in a further question asking women about their willingness to participate in possible treatment trials. After the first questionnaire iteration it became evident that women wanted to explain why they answered ‘No’ or ‘Unsure’ to participating in the trials. |
| **Overlapping response categories** | In two questions relating to what women thought triggered their first and recurrent episodes of BV, we found that women ticked multiple response boxes to account for the same trigger i.e. ‘sex with a male partner’ and ‘sex with a new partner’ to represent ‘sex with a new male partner’ or ‘sex without a condom’ and ‘penile vaginal sex’ to represent ‘unprotected penile vaginal sex’. The categories were revised to minimise cross over in response options. An additional question was also inserted following each of these two questions to allow participants a free text area in which to provide further explanation on why they thought certain actions or behaviours had triggered their first or recurrent episodes of BV. |
